# Promoter switching in response to changing environment and elevated expression of protein-coding genes overlapping at their 5’ ends

**DOI:** 10.1038/s41598-021-87970-w

**Published:** 2021-04-26

**Authors:** Wojciech Rosikiewicz, Jarosław Sikora, Tomasz Skrzypczak, Magdalena R. Kubiak, Izabela Makałowska

**Affiliations:** 1 Center for Applied Bioinformatics, St. Jude Children’s Research Hospital, Memphis, TN USA; 2 Institute of Human Biology and Evolution, Faculty of Biology, Adam Mickiewicz University, Poznań, Poland; 3 Center for Advanced Technology, Adam Mickiewicz University, Poznań, Poland

**Keywords:** Gene expression, Gene regulation, Transcription

## Abstract

Despite the number of studies focused on sense-antisense transcription, the key question of whether such organization evolved as a regulator of gene expression or if this is only a byproduct of other regulatory processes has not been elucidated to date. In this study, protein-coding sense-antisense gene pairs were analyzed with a particular focus on pairs overlapping at their 5’ ends. Analyses were performed in 73 human transcription start site libraries. The results of our studies showed that the overlap between genes is not a stable feature and depends on which TSSs are utilized in a given cell type. An analysis of gene expression did not confirm that overlap between genes causes downregulation of their expression. This observation contradicts earlier findings. In addition, we showed that the switch from one promoter to another, leading to genes overlap, may occur in response to changing environment of a cell or tissue. We also demonstrated that in transfected and cancerous cells genes overlap is observed more often in comparison with normal tissues. Moreover, utilization of overlapping promoters depends on particular state of a cell and, at least in some groups of genes, is not merely coincidental.

## Introduction

The presence of protein-coding genes located on opposite strands of DNA and sharing fragments of genomic sequences in a sense-antisense orientation (i.e., overlapping genes) was reported in mammalian genomes over 30 years ago^1^. In subsequent years, numerous studies have shown that the existence of sense-antisense gene pairs is notably widespread in the genomes of humans and other species^2–5^. Nevertheless, with reports describing the common occurrence of noncoding RNAs transcribed from the opposite strands of protein-coding genes^6, 7^, interest in sense-antisense pairs of two protein-coding genes has significantly decreased. However, recent advances in technologies and sequencing approaches have resulted in an unprecedented outburst of novel data, which provides a great opportunity to examine sense-antisense protein-coding genes in a new way and reinvestigate the phenomenon of this very specific case of antisense transcription.

Sense-antisense gene pairs may be classified by their mutual positions into several categories, as described elsewhere^2^, and may overlap within exonic and/or intronic regions. Overlaps involving exons of both genes form so-called *cis*-natural antisense transcripts (NATs). This term is often reserved for noncoding RNA transcribed from the opposite strands of protein-coding genes. Since this paper is dedicated to sense-antisense pairs of protein-coding genes, the term “overlapping genes” will be employed to avoid any confusion.

Over time, many approaches for overlapping gene detection have been developed, and each approach resulted in a different number of identified genes. A relatively simple method for identification of the overlapping genes was applied by Lehrer et al.^8^, who utilized the BLAST program to identify complementary regions in mRNA sequences. This approach enabled the researchers to identify 61 tail-to-tail, 20 head-to-head and 4 nested overlapping gene pairs in the human genome. In other methods, expressed sequence tag (EST) libraries, as well as mRNA sequences, were used^9^. In these studies, 144 human and 73 mouse NATs were identified. Veeramachaneni et al.^2^ used annotated human and mouse genomic sequences from GenBank to identify genes with overlapping coordinates, identifying 774 and 578 overlapping gene pairs in the human and mouse genomes, respectively.

More recently, Conley and Jordan^10^ proposed a different approach for NAT identification. Focusing on antisense transcriptional start sites in the promoter regions of all annotated genes, these researchers discovered thousands of antisense transcription start sites (TSSs). The researchers also identified overlapping genes with so-called active and weak promoters using cap analysis of gene expression (CAGE) and chromatin immunoprecipitation followed by sequencing (ChIP-seq) data from the ENCODE project^11^. This analysis has also shown tissue-specific expression patterns, suggesting a functional role of the gene overlap phenomenon. Ling and coworkers^12^, using commercial oligonucleotide DNA microarrays, have also identified thousands of NATs and showed tissue-specific overlapping transcripts in a set of 9 analogous human, mouse and rat cell lines. Although these two more recent studies investigated all *cis*-NATs, not only protein coding genes, they demonstrate the potential of new technology applications to study sense-antisense gene pairs.

Many functions of the gene overlap phenomenon have been suggested since they were first reported; however, its significance remains a matter of debate^13^. Nevertheless, a wide variety of biological roles have been assigned to many natural antisense transcripts^14, 15^. Faghihi and Wahlestedt^14^, in their review paper, presented the division of these functions into three main categories: transcription interference, DNA–RNA interactions and RNA–RNA interactions. Transcriptional interference (TI) is the direct or indirect inhibitory influence of one transcriptional process on another. An example of direct TI is RNA polymerase (RNAP) collision, which can occur when the transcription of head-to-head overlapping genes takes place at the same time. Other TI examples include promoter competition, so-called “sitting duck” interference or occlusion^16^. Transcripts of overlapping genes may also regulate transcription at the RNA–DNA interaction level. Examples of such phenomena could be DNA methylation and demethylation or downregulation of the expression of the sense gene by antisense RNA^17^. Chromatin modification and silencing of the sense promoter have also been demonstrated^18^. The complementarity of two antisense transcripts may also lead to RNA–RNA interactions by the formation of sense/antisense RNA duplexes^19^. As shown by Hastings et al.^20^, such a duplex may physically hide access to splicing sites, which may result in the formation of alternative splice forms. RNA-RNA duplexes may also have an influence on transcript transport^21^, contribute to endo-siRNA^22^, or have a stabilizing effect on protein-coding sense transcripts by blocking the RNA destabilizing motif^23^or by competing for microRNA sites^24^. Chen et al.^25^, in support of antisense regulation by forming dsRNA, showed that human sense–antisense transcripts tend to be coexpressed and/or inversely expressed more frequently than expected by chance. Henz et al.^26^ also observed negatively correlated expression of overlapping transcripts in *Arabidopsis thaliana*. On the other hand, Jen et al.^27^ argued that although there is a very high level of joined expression of sense and antisense transcripts in *A. thaliana,* detailed analysis of microarrays did not imply any dsRNA-based transcript degradation. The study of human and mouse transcripts performed by Osato et al.^28^ supports the transcriptional collision model by showing that the expression level of antisense transcripts decreases as the length of the overlap region increases.

Despite the number of studies focused on sense-antisense transcription, the key question of whether such organization evolved as a regulator of gene expression or if this is only a byproduct of other regulatory processes has not been elucidated to date. In this study, a specific group of genes in sense-antisense orientation, i.e., protein-coding antisense gene pairs were analyzed with a particular focus on pairs overlapping at their 5’ ends. Analyses were performed in 73 human TSS libraries. The obtained results demonstrate that the gene overlap is poorly conserved and quite often tissue-specific, but most notably, the overlap at the 5’ ends of genes is an unstable feature, and the role of this type of gene overlap may be highly complex. In addition, analysis of expression level did not demonstrate any negative effect of gene overlap. In contrast, genes utilizing overlapping TSSs have, on average, higher expression levels. This finding prompted us to search for possible mechanisms enabling to escape from adverse consequences of potential transcriptional interference. As a putative mechanism, we investigated monoallelic expression (MAE).

## Results

### Identification of 5’ overlapping genes

Transcription start site data from 73 human tissues and cell lines were analyzed to identify 5’ overlapping genes. Samples included libraries from healthy adult tissues and organs, fetal tissues, cell lines in various experimental conditions and lung cancer cell lines. A total of 15,778 genes had at least one TSS assigned in one or more analyzed libraries. List of libraries, the number of TSSs identified in each library and the corresponding gene numbers are provided in Supplementary Table S1.

**Figure 1:**
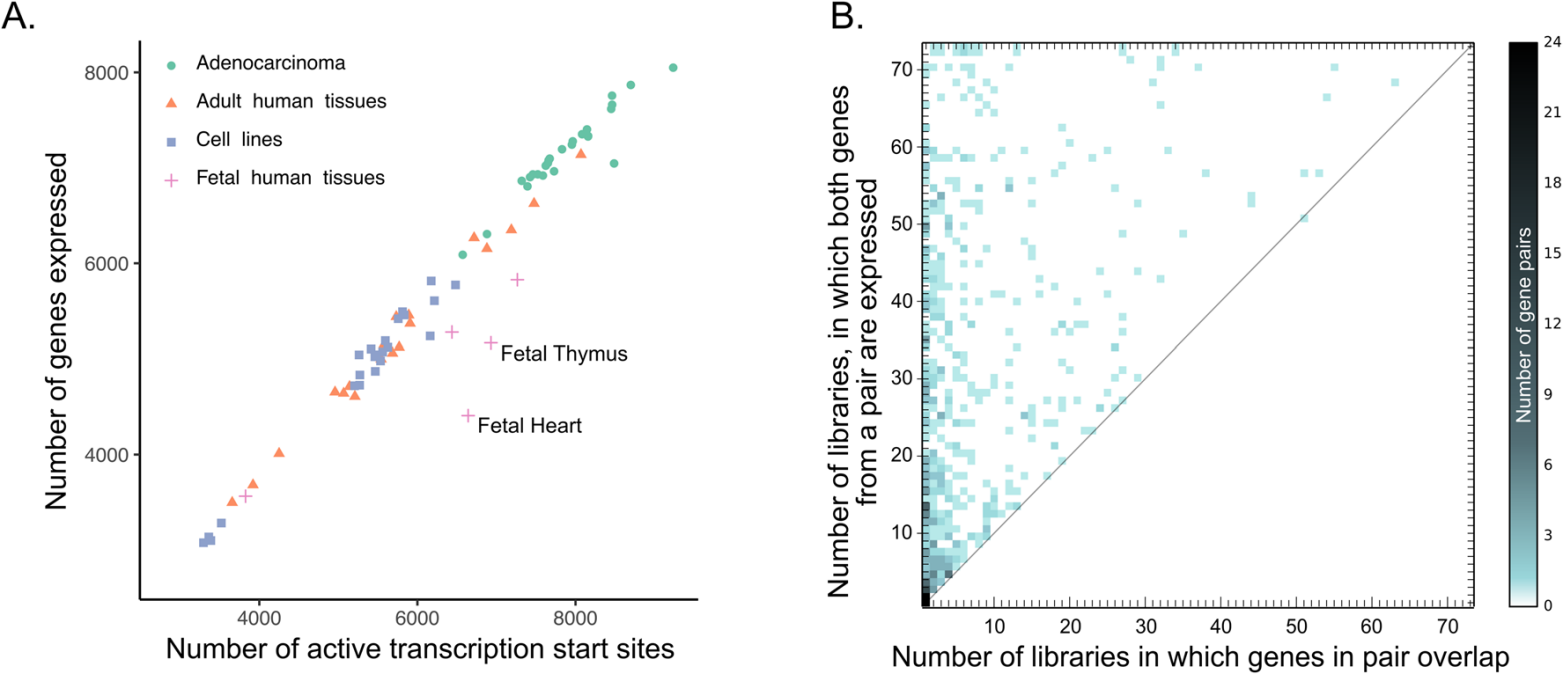
(**A**) Correlation between gene number and the number of transcription start sites. (**B**) Relation between the number of libraries in which genes from the given pair are expressed and the number of libraries in which they overlap.

Most of the genes, that is, approximately 57% had only one, although not always the same, TSS in every library that the gene was expressed in. Genes that consistently used multiple promoters, reflected by multiple TSSs, were relatively rare. Only 106 genes fall into this category. The number of TSSs identified in each library, as expected, was highly correlated with the number of expressed genes (Fig. 1A). Interestingly, the average number of TSSs per gene was always higher in fetal tissues than in corresponding adult tissues (Supplementary Table S1). For example, in the adult thymus, the mean number of TSSs per gene is 1.17, while in the fetal thymus, it is 1.34. This difference is exceptionally visible in the case of the heart, where in adult tissue, the average number of TSSs per gene is 1.04, and in the fetal heart, the number is 1.51. These data clearly demonstrate that genes utilize a notably higher number of alternative promoters in fetal tissues than in adult tissues. The gene with the highest number of assigned TSSs, gene *RYR2*, in fetal heart utilizes as many as 115 TSSs.

The coordinates of genes and assigned to them TSSs were analyzed, and genes were considered “overlapping” if the overlap of at least 1 bp was detected at their 5’ end ends in at least one library. Altogether, 582 pairs of genes overlapping at their 5’ ends were identified in the human genome. The majority of gene pairs overlapped only in selected libraries, and no single pair was expressed and overlapped in all of them (Fig. 1B). 52 gene pairs overlapped in every library in which both genes were expressed (genes on diagonal line in Fig. 1B). Among those genes, *ATF5-NUP6* was expressed in 51 libraries and always used overlapping TSSs. Ten pairs were expressed in all 73 libraries; however, they overlapped only in some of them (genes on the top line on Fig. 1B). The observation that most genes overlap only in selected libraries is associated with the known fact that many genes utilize multiple promoters and TSSs^29–31^ that could be used individually or in certain combinations^32, 33^. Although our results may not be very surprising**,** we demonstrated for the first time that the switch from one promoter to another may lead to changes in gene arrangements, i.e., from overlapping to not overlapping and vice versa.

**Figure 2:**
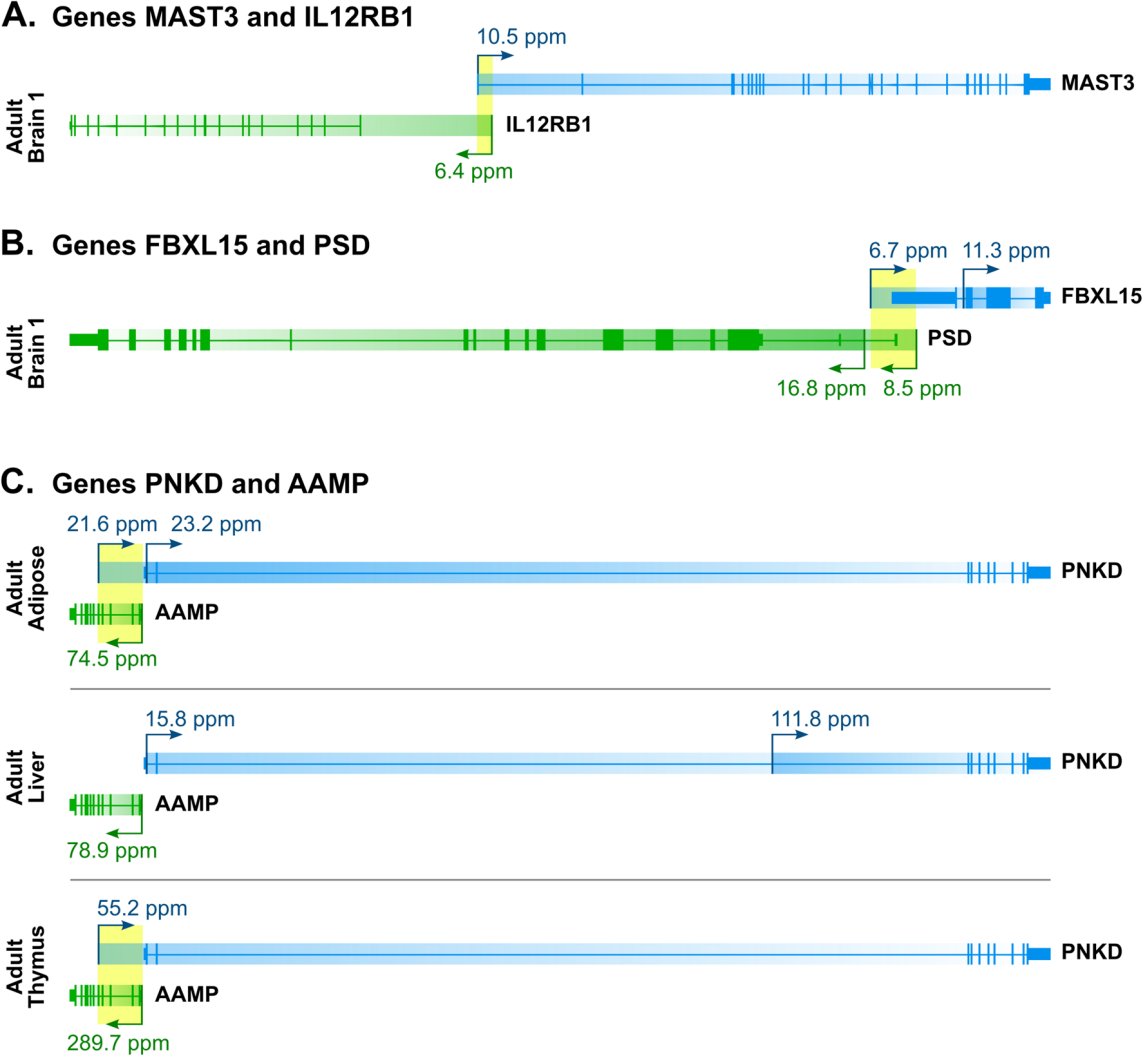
(**A**) Genes *MAST3* and *IL12RB1* expressed exclusively from single and overlapping TSSs. (**B**) Both *FBXL15 and PSD* use overlapping and not overlapping TSSs. (**C**) Gene *PNKD* demonstrates that variation in TSS use depends on the library, resulting in various overlap statuses of the *PNKD-AAMP* gene pair.

Among all analyzed genes 506 used multiple promoters in at least one library, in which they overlapped. Of these genes, only 7 use exclusively overlapping TSSs (example shown in Fig. 2A). In the remaining instances, overlapping TSSs were used together with nonoverlapping TSSs (Fig. 2B). Therefore, the status of a given pair, overlapping or nonoverlapping, depends on the combination of TSSs utilized in a given tissue. In the example of the genes *PNKD* and *AAMP* (Fig. 2C), overlap is present in two tissues, adult adipose and fetal thymus. In the fetal thymus, all transcripts start from overlapping TSSs. In adult adipose tissue, however, less than half of *PNKD* transcripts utilize overlapping TSSs. Thus, the fact that a given gene has overlapping TSS does not necessarily mean that all mRNAs are transcribed from overlapping start sites.

### Comparison of 5’ overlapping genes across human samples

**Figure 3:**
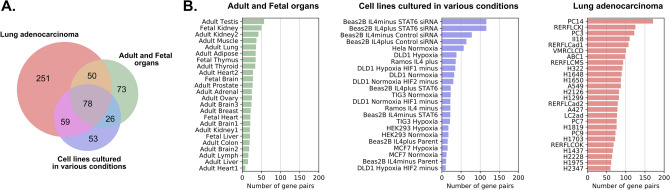
(**A**) Venn diagram showing the number of overlapping gene pairs shared between three analyzed data sets. (**B**) Numbers of overlapping gene pairs in the subsets of samples.

The expression pattern, as well as utilization of alternative promoters, may depend on tissue-specific factors, environmental conditions and the genotype/phenotype of a given individual. These might lead to cross-sample variation in the utilization of overlapping or nonoverlapping promoters. Indeed, as shown on the Fig. 3, the number of overlapping genes significantly differs between samples. To look closer at factors that may influence this variation, we divided all human samples into three groups: healthy tissues and organs, cell lines in various experimental conditions and lung adenocarcinomas. For this particular analysis, the HeLa cell line was excluded from the second group as it was cultured in only one experimental condition. The highest number of 5’ overlapping genes (430) was identified in lung adenocarcinoma samples (Fig. 3A). The largest variation in the number of overlapping gene pairs was observed in cell lines cultured in various conditions (Fig. 3B). Interestingly, out of 580 gene pairs that overlapped in at least one of 72 analyzed libraries, only 80 pairs overlapped in at least one library in each set. Many overlapping pairs were observed exclusively in a specific data set; for example, as many as 241 pairs overlapped only in adenocarcinoma (Fig. 3A).

**Figure 4:**
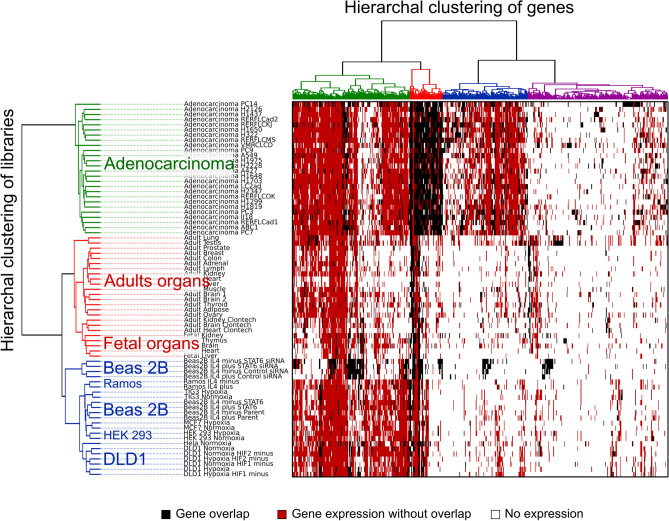
Hierarchical clustering of overlapping gene pairs in 73 samples.

Considering that only a small fraction of genes overlapped in at least one sample from each set, hierarchical clustering of all overlapping genes was performed to test whether the variation in the overlap between genes could have some functional meaning or if it represents only a random property. As shown in Fig. 4, libraries are clearly grouped into three large clusters perfectly reflecting three sample groups. In addition, in the cell line group, transfected BEAS-2B cell lines (top four cell lines in the blue library cluster) significantly differed from other samples. Gene clustering also demonstrated a very distinctive cluster of genes overlapping almost exclusively in adenocarcinoma (red cluster of genes in Fig. 4). A few smaller clusters of gene pairs that are expressed in various samples but with a high propensity to overlap in four transfected BEAS-2B cell lines were also detectable.

**Figure 5:**
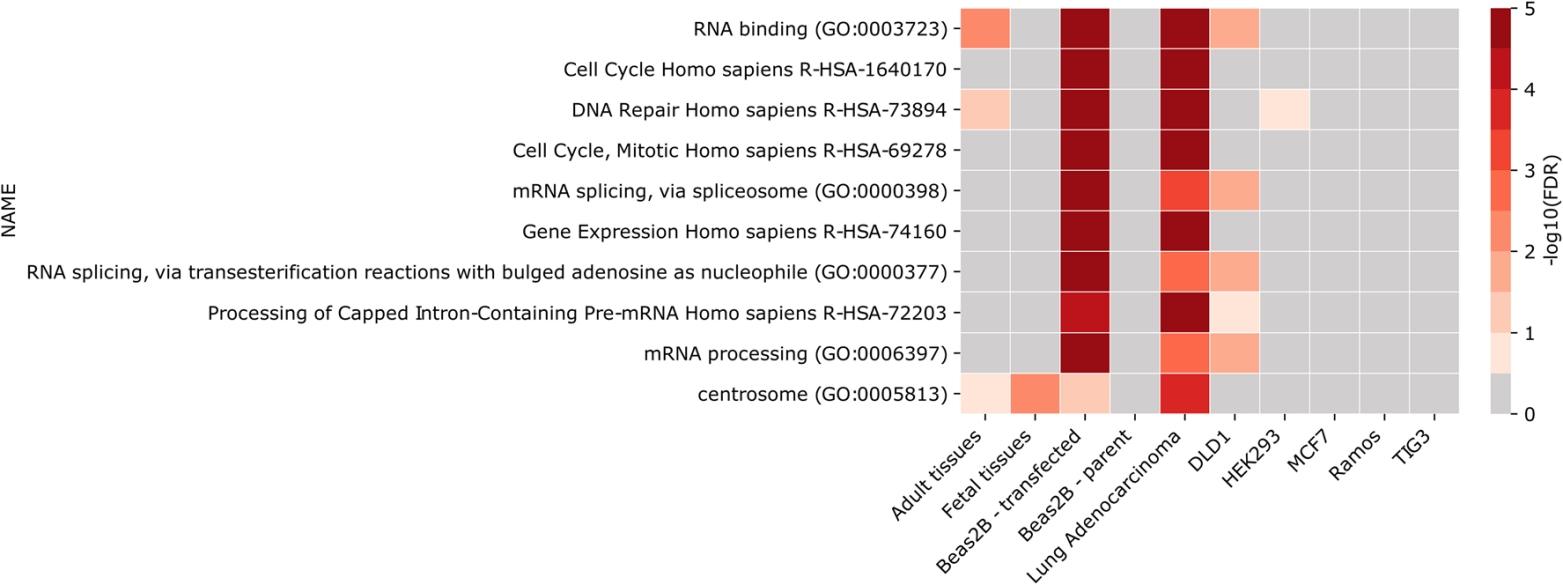
Enrichment of top 10 gene signatures among overlapping genes from grouped libraries.

Hierarchical clustering demonstrated that utilization of overlapping promoters is not, at least in some groups of genes, simply incidental. These results motivated us to investigate if the genes that do overlap in particular groups of libraries are enriched with specific gene signatures. Interestingly, in the group of genes overlapping in at least one adenocarcinoma, as well as genes overlapping in transfected Beas2B cell lines, observed is the largest number of overrepresented signatures. Here, pathways such as RNA binding, regulation of mRNA splicing and processing or the genes related to DNA Repair, are among the most strongly enriched (Supplementary Table S2, Fig. 5). Some signatures are also increased in colorectal adenocarcinoma cell lines (DLD1). On the other hand, adult and fetal tissues, as well as other cell lines, are in comparison sparsely enriched in any specific gene signatures (Supplementary Table S2, Fig. 5). In combination, these results straighten the conclusion, that gene overlap is not a stochastic event.

### Expression of 5’ overlapping genes

It was proposed that the major role of gene overlap is the fine-tuning of their product levels^16^. It was also suggested that sense-antisense transcripts tend to be coexpressed and often have anticorrelated expression^25, 26^. More recent works, however, demonstrated that sense/antisense transcripts, at least in the case of lncRNA-protein coding pairs, tend to have positively correlated expression^12^. To determine which of these two concepts, if any, applies to this special case of protein-coding sense/antisense gene pairs, 73 pairs that overlap in at least 10 libraries and do not overlap in a minimum of 10 other libraries were selected and analyzed. Pearson’s correlation coefficient of the expression level was calculated for each pair in libraries where selected genes overlap and separately where they do not (Supplementary Table S3). Overall, we observed 33 cases of positive expression correlation. Twenty-one pairs had positive correlations only in libraries in which they overlapped, four pairs had positive correlations only in libraries in which they were nonoverlapping and eight pairs in both. A significant negative correlation was observed only in three instances, that is, once when the genes overlapped and twice in the case of nonoverlapping pairs.

**Figure 6:**
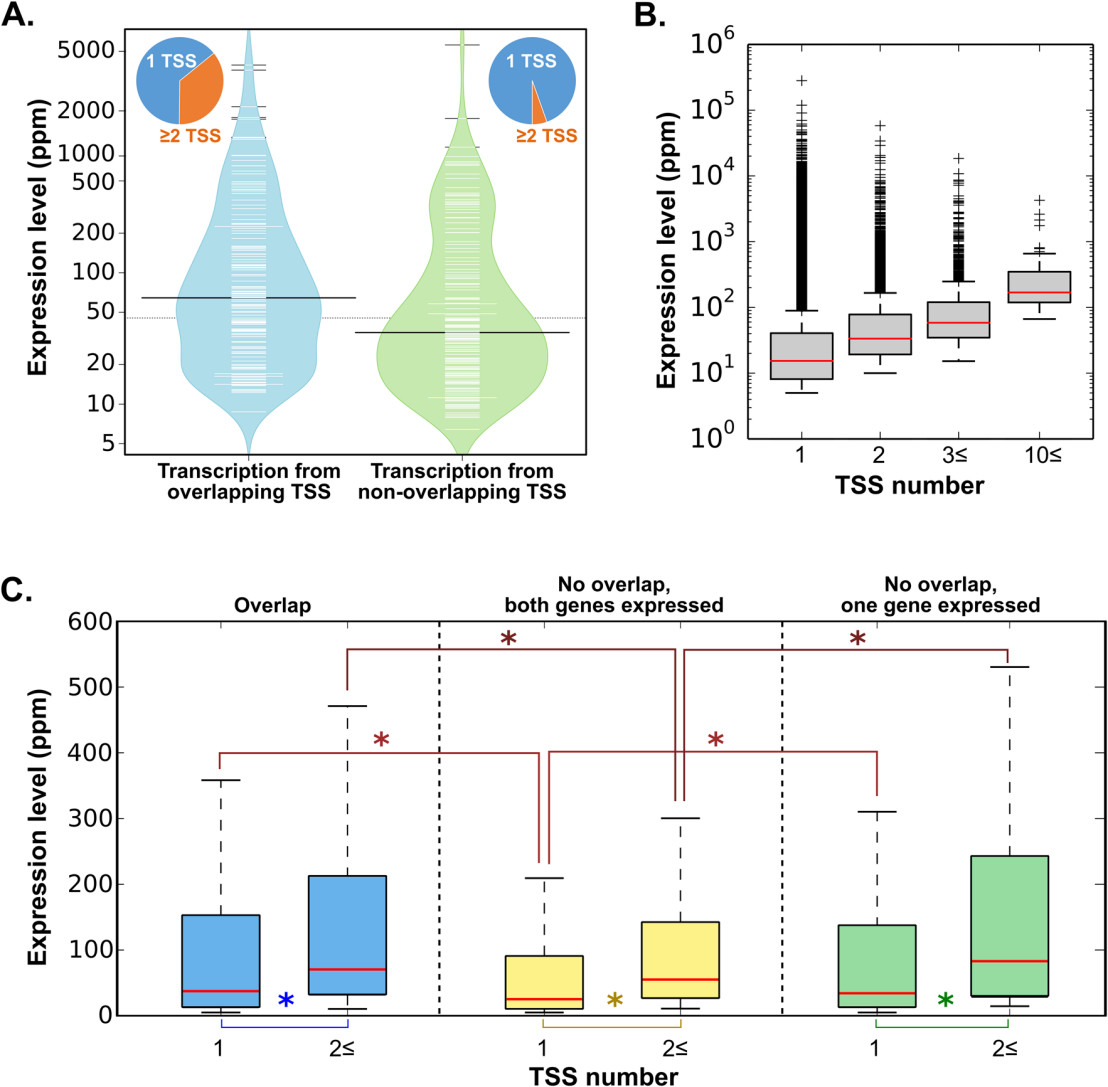
(**A**) Expression level of 73 pairs of genes in libraries in which they overlap and those in which they do not overlap. (**B**) Expression of all genes in all libraries divided according to the number of utilized TSSs. (**C**) Expression of genes from 73 analyzed pairs according to their expression, genomic arrangements and number of utilized TSSs. Statistically significant differences are marked by an asterisk (*).

Although a negative expression correlation was observed very rarely, we cannot exclude the possibility that overlap, due to potential polymerase collisions, negatively influences the expression of both involved genes. To verify this hypothesis, a paired t-test was performed to compare the average expression levels of genes from the same 73 pairs in libraries in which they overlap and in those in which they do not. Analysis showed that these genes have, on average, higher expression levels when they are utilizing overlapping TSSs, and the difference is significant at *p* ≤ 0.05 (Fig. 6A).

In the course of the analysis, it was noticed that genes from 73 selected pairs frequently used multiple promoters while they overlapped. Based on this observation, it could be hypothesized that when more product is required, additional TSSs are employed to increase the efficiency of the transcription, although this leads to gene overlap. To verify the hypothesis that the utilization of multiple TSSs results in higher expression, the expression of all genes in all 73 human libraries was analyzed. Genes were grouped according to the number of utilized TSSs, and indeed, the expression level was, on average, higher for genes that utilized more TSSs (Fig. 6B). The observed differences were determined to be significant (Mann–Whitney U test ≈ 0). Therefore, in the next step, genes from the abovementioned 73 pairs were divided into six categories according to their organization and the number of TSSs used in each respective library (Fig. 6C). The expression of genes in each category was calculated, and the results of the analysis demonstrate that genes utilizing multiple TSSs have higher expression levels regardless of their genomic organization: (1) genes overlap, (2) genes do not overlap or (3) only one gene from the pair is expressed (Fig. 6C). These differences were significant (Mann–Whitney U test). Interestingly, genes that were not overlapping had significantly lower expression levels in comparison with overlapping pairs or when only one gene from a pair was expressed. This was true for genes utilizing a single TSS, as well as genes with multiple TSSs. This demonstrates that although the number of utilized TSSs influences the expression level, some other factors contribute to the lower expression of genes, while they utilize nonoverlapping TSSs. This observation contradicts earlier suggestions that transcriptional interference negatively influences the expression of overlapping genes^28^.

### Monoallelic expression as a putative mechanism of escaping from transcriptional interference

There are a number of possible mechanisms that could potentially “protect” overlapping genes from downregulatory effect of transcriptional interference. One of these mechanisms, a highly plausible one, is monoallelic expression^34^. In such cases, overlapping genes would be physically separated from each other; hence, simultaneous transcription of both genes would not cause TI. To test this hypothesis, analysis of genomic (WGS) and transcriptomic (RNA-seq) data from 22 adenocarcinoma libraries was performed. Of 413 gene pairs overlapping in at least one of these libraries, 295 pairs met the requirements of the minimal expression level of 5 FPKM in at least one library. This condition was also met by 108 genes, where only one gene from a given pair was expressed in a particular library. Altogether this gave 698 genes that were further analyzed.

**Figure 7:**
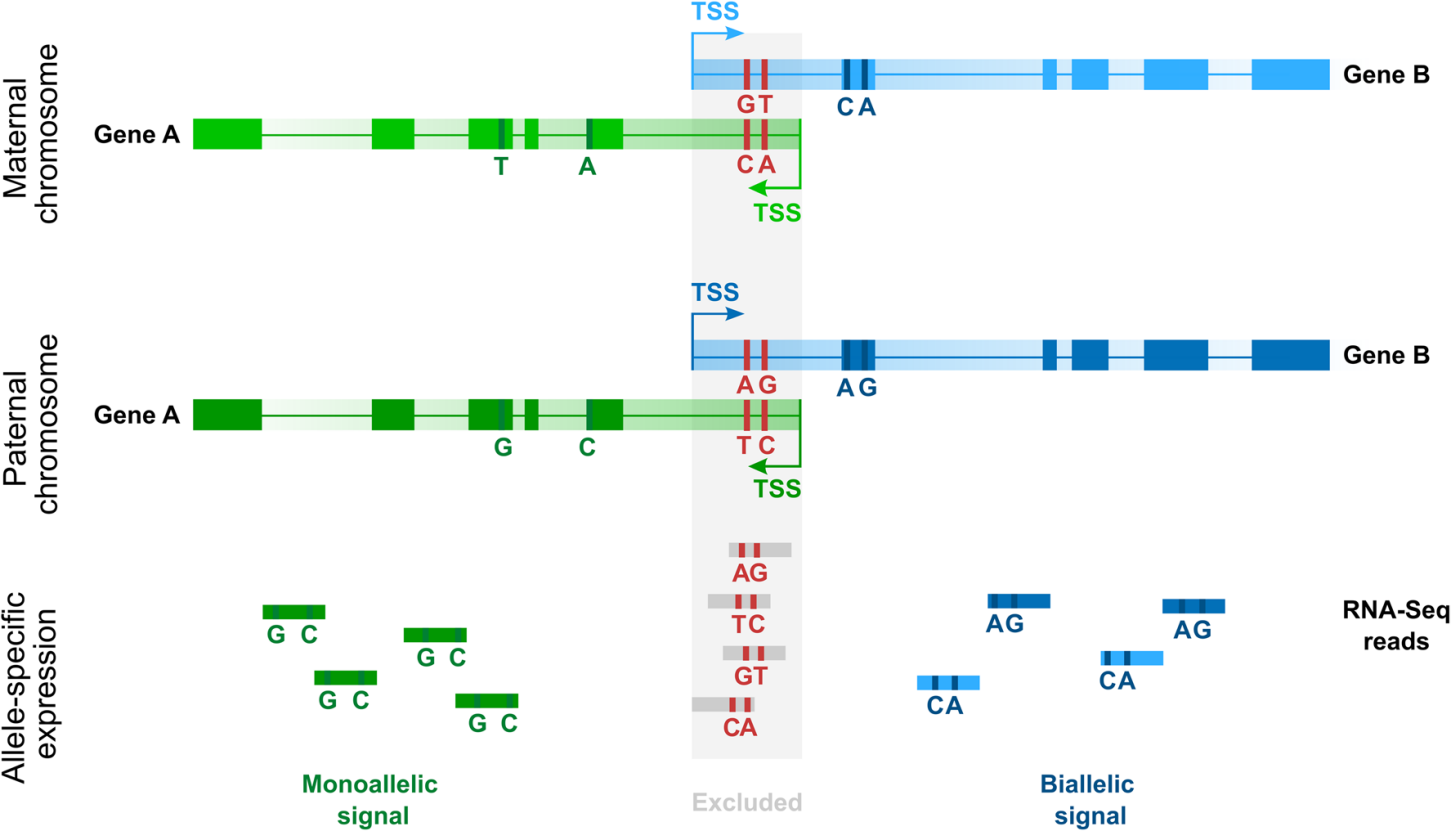
Signals from overlapping regions may indicate biallelic expression even in the case of allele-specific expression of both genes.

SNP analysis was performed after excluding overlapping regions, as they may exhibit biallelic expression signals even when each gene is expressed from different chromosome from a homologous pair, as demonstrated in Fig. 7. This analysis identified polymorphic sites in 83 pairs and 22 single genes. Based on the RNA-seq data, a ratio of allele expression was estimated, and genes were categorized as monoallelic, biallelic, skewed or noninformative according to rules described in Materials and Methods. In the case of 20 genes, the results were noninformative, since some polymorphic sites gave monoallelic and other biallelic or skewed signals. In the remaining set detected were 111 genes with biallelic expression, 10 genes with monoallelic expression and 7 skewed. In the instance of 40 genes, the expression pattern differed between libraries, i.e., in some libraries, expression was biallelic, and in others, it was monoallelic or skewed (Supplementary Table S4).

Data obtained for overlapping genes were compared with the expression pattern of 4642 protein-coding genes that do not overlap and in at least one library have expression at or above 5 FPKM. Of these genes, for 1718 genes, polymorphic sites were not detected, 1111 genes always exhibited biallelic signals, and 108 genes always exhibited monoallelic expression. The remaining genes showed either mixed or skewed expression.

The number of genes with monoallelic expression in both sets is notably low and may be underestimated due to the lack of polymorphic sites in the majority of analyzed cases. Nevertheless, the ratio of genes with clear monoallelic expression to genes with biallelic expression was largely the same (close to 1:10) in the overlapping gene set and in the control. These results indicate that although some overlapping genes do have monoallelic expression, it is most likely not a major mechanism helping to avoid the risk of transcriptional interference.

### Effect of transfection on TSSs in a normal lung epithelial cell line: case study

A number of constant changes in response to experimental factors were observed in BEAS-2B (bronchial epithelial) cell lines. Transfection with STAT6 silencing siRNA of both IL4 (interleukin 4) plus and IL4 minus BEAS-2B cell lines^35^ resulted in a rapid increase in the number of 5’ overlapping gene pairs (Fig. 3B). While, for example, in the cell line ‘BEAS-2B IL4 minus STAT6 siRNA’, there are 116 overlapping gene pairs, there are merely twelve in the corresponding nontransfected control ‘BEAS-2B IL4 minus the parent’ line. Similar observations can be made in the case of transfection with nontargeting siRNAs (BEAS-2B IL4 plus/minus Control). Overall, as many as 15 gene pairs changed their status from being nonoverlapping in both BEAS-2B parent (not transfected) cell lines to overlapping in all four BEAS-2B transfected cell lines.

**Figure 8:**
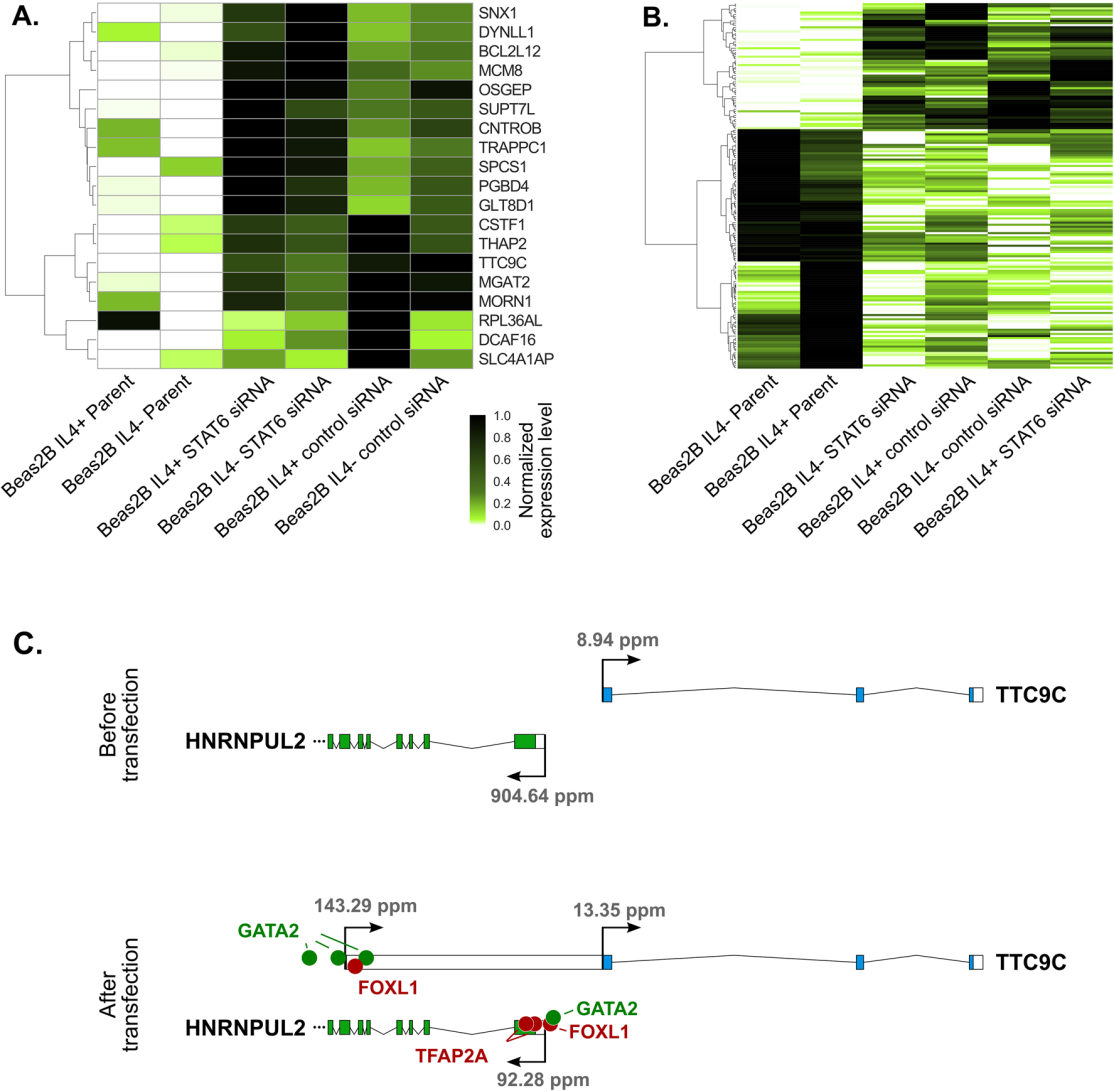
(**A**) Heat map representing the expression of 19 genes that switched the TSS in four transfected BEAS-2B cell lines. (**B**) Heat map representing differentially expressed transcription factors. (**C**) Changes in TF expression after transfection in a case study of the *TTC9C* and *HNRNPUL2* pair. Green dots represent binding sites for upregulated transcription factors, and red dots represent downregulated transcription factors.

These 15 pairs of genes that switched from nonoverlapping to overlapping status in the response to transfection were further investigated. In the majority of these cases, that is, 11 out of 15, the change in the TSS was observed only in one gene from a pair and, in four cases, in both of them. Altogether, 19 genes shifted to or additionally activated upstream located TSSs. Differential expression analysis demonstrated that all 19 genes increased their expression after the switch to a more distant TSS (Fig. 8A). In the case of seven genes, *MGAT2, TTC9C, OSGEP, DCAF16, SUPT7L, PGBD4* and *GLT8D1*, the change was statistically significant. We hypothesized that the promoter switch and the increase in the expression level could be induced by changes in the expression of transcription factors resulting from transfection. To investigate this possibility, the expression of transcription factors in two nontransfected and four transfected BEAS-2B cell lines was compared. Indeed, 174 transcription factors significantly changed the expression in the reaction to transfection with STAT6 silencing siRNA and with nontargeting siRNA; 60 genes were upregulated, and 114 were downregulated (Fig. 8B). To investigate whether any of these transcription factors could regulate genes that in response to the transfection changed TSS, the analysis of transcription factor binding sites in the region of 500 bp upstream and downstream of TSSs was performed using the JASPAR database^36^. For 18 genes (out of 19 analyzed), the binding site for at least one of the differentially expressed TFs was observed in the proximity of one or both associated TSSs.

For some genes, such as the gene *TTC9C* encoding tetratricopeptide repeat (TPR) protein 9C, which overlaps with the *HNRNPUL2* gene encoding Heterogeneous Nuclear Ribonucleoprotein U-Like Protein 2, the TSS switch may indeed be explained by changes in transcription factor expression. These two genes do not overlap in lung epithelial BEAS-2B parent (i.e., not transfected) cell lines. However, after transfection, an additional TSS was activated for the *TTC9C* gene. Consequently, these two genes changed from nonoverlapping to overlapping (Fig. 8C). For the *TTC9C* gene, we identified TSS-specific (i.e., present in the proximity of only one TSS out of two used) binding sites for two transcription factors, GATA2 and FOXL1, that changed expression levels after transfection (Fig. 8C). GATA2, which was shown to be an activator of the number of genes^37, 38^, was upregulated after transfection and was associated with TSS activation in response to transfection. On the other hand, the transcription factor FOXL1, which is known to act as a repressor^39, 40^, was downregulated. These two changes could contribute to the fact that an additional promoter was activated and, as a consequence, the transcription of *TTC9C* increased. Interestingly, the expression of the *HNRNPUL2* gene was decreased tenfold after activation of overlapping TSS. This phenomenon could be an effect of the overlap; however, analysis of 34 libraries in which these two genes overlap, as well as 32 libraries where they do not, did not demonstrate any expression correlation, regardless of the genomic arrangement. The decrease in the expression of *HNRNPUL2* could also be caused by changes in the expression level of TFs, including the downregulation of activating enhancer binding protein 2α (TFAP2A), or by some other factors. Clearly, in the case of both genes, the regulatory role of pinpointed factors, as well as the possible impact of the overlap, are only hypothetical and should be verified experimentally.

## Discussion

In the majority of previous studies, the identification of protein-coding genes in sense-antisense orientation was performed without considering the type of tissue or cell line from which the mRNA was isolated. Therefore, genes were simply classified as overlapping, i.e., forming *cis*-natural antisense transcripts, or as nonoverlapping. However, it is known that many genes, especially those with CpG-rich promoters, can use alternative TSSs and, as a result, produce transcripts with different lengths at their 5’ ends^41, 42^. The presence of alternative TSSs is highly common for the majority of human genes^41, 43^, and more than one TSS from a single gene can be utilized at the same time point^44^. Tan et al.^45^ found that 35% of examined human erythroid genes had alternative first exons and promoters, and Kim et al.^29^ identified 1609 genes (24%) with active multiple promoters in human fibroblast cells. In our dataset (i.e., all genes expressed in at least one library), approximately 57% of genes always utilized only one TSS, although not necessarily the same one. Genes that consistently used multiple promoters, as indicated by multiple TSSs, were relatively rare. Only 106 genes in humans fall into this category. Interestingly, the average number of utilized TSSs was always higher in fetal tissues than in the corresponding adult tissues. This finding is in keeping with the results obtained by Beak et al., who found overrepresentation of alternative promoters among genes involved in development^46^. The analysis performed by these researchers indicates that alternative promoters are more abundantly utilized in the brain, heart, liver and related tissues in the embryonic and fetal stages. Studies on *Drosophila*^47^ demonstrated that over 40% of developmentally expressed genes have at least two promoters.

In this study, we analyzed TSS data from 73 different human libraries to investigate whether the switch in utilization of alternative TSS could lead to changes in the gene status from overlapping at the 5’ end to nonoverlapping and vice versa. Analysis led to the identification of 582 pairs of protein-coding genes that overlapped at their 5’ ends in at least one of the analyzed samples. This number is higher than those obtained in some previous studies. Veeramachaneni et al.^2^, for example, identified only 243 head-to-head oriented overlapping gene pairs in the human genome. However, this difference can be easily explained by the limited quantity of data available at that time. On the other hand, we identified considerably fewer pairs than in several more recent works. In the FANTOM3 project, for instance, researchers identified as many as 1638 sense-antisense pairs. However, they also examined overlaps between protein-coding and ncRNA genes^3^. In the set of pairs of overlapping genes identified by our group, we did not identify any that overlapped in all 73 human libraries, probably because potentially overlapping genes are not expressed in all libraries. This finding is interesting and might suggest that housekeeping genes are not involved in overlaps. Fifty-two pairs of genes overlapped in every library in which both genes were expressed. Of these pairs, one pair, *ATF5-NUP62*, was expressed and always overlapped in 51 libraries, and an additional three pairs, *FAM120A-FAM120OS*, *CIAO1-TMEM127*, and *PPCS-ZMYND12*, were expressed and overlapped in 27, 26 and 23 libraries, respectively. The remaining pairs that were observed to always utilize overlapping TSSs were expressed in only a few or only one library.

While studying the chromatin environment and RNA Pol II binding properties of human *cis*-NAT promoters, Conley and Jordan^10^ found that the number of *cis*-NATs across 16 cell types significantly differs. In our studies, which focused on *cis*-NATs formed between two protein-coding genes, we also observed differences in the number of 5′ overlapping genes among analyzed tissues and cell lines. These discrepancies may be attributable to either a lack of expression of one or both genes from a given pair in a particular tissue or from the switch to an alternative promoter (see Fig. 6C). The utilization of particular TSSs may be affected by multiple factors. To examine these factors more closely, human overlapping genes were analyzed in three separate datasets: normal tissues, cell lines cultured under various conditions and adenocarcinoma cell lines from tumors obtained from 26 patients. Our data show that utilizing overlapping TSSs is tissue-specific. Ling et al.^12^ also identified tissue-specific overlapping transcripts in humans, mice and rats. In addition to tissue-specific components, our study demonstrates that other factors, such as environmental conditions and genetic/phenotypic backgrounds, also contribute to the switch from overlapping to nonoverlapping gene arrangement and vice versa. Hierarchical clustering of all overlapping genes, as well as the reaction to the transfection that leads to TSS switching and gene overlaps, demonstrates that the observed differences among samples are not purely accidental. There is clearly a group of genes that preferably use overlapping TSSs in adenocarcinoma, while another group of genes favors overlapping TSSs in transfected cells. Nineteen genes were observed to respond to the transfection in exactly the same way in all four transfected cell lines and were determined to switch to more distant promoters that led to the overlap of genes. The activation of upstream promoters in reaction to changing environments has been demonstrated previously. For example, Singer et al.^48^ identified 25 genes that activated upstream promoters after estrogen stimulation of MCF7 cells. Nevertheless, to the best of our knowledge, alternative promoter activation and inactivation had not been studied previously in the context of switching between overlap/nonoverlap status in a given gene pair.

The observation that there is increased number of overlapping genes in adenocarcinoma and in Beas2 transfected cell lines motivated us to investigate functions of these genes. Analysis of genes signatures revealed that in tissues or cell lines with significant changes in the internal environment, either due to transfection or cancer, some categories are enriched in sets of genes utilizing overlapping TSSs. Especially overrepresented are genes related to regulation of splicing, DNA repair, RNA binding or cell cycle. It is known that cancer cause disturbance in the cell homeostasis^49^. Similarly, introducing an exogenous material during transfection may generate stress in a cell. Therefore, the observation could be either a byproduct of reprogrammed cell metabolism or may be related to activation of some defense mechanisms. Either way, it demonstrates that alterations in the cell homeostasis may prompt significant changes in the use of alternative promotors and lead to overlap of genes at their 5′ ends.

Changes in alternative TSS usage are not only observed at the “on/off’ level. The ratio of expression from overlapping and nonoverlapping TSS also fluctuates, and the pattern varies from gene to gene. Because the overlap between genes may cause transcriptional interference, one may expect that overlapping TSSs would be the minor ones and that transcription of the majority of genes would be initiated at nonoverlapping, rather than overlapping, promoters. Indeed, in some cases, transcription from downstream TSSs dominates whenever overlapping and nonoverlapping TSSs are utilized. For example, according to our OverGeneDB database^50^, in the case of the *C11orf48 gene,* which overlapped with *UQCC3* in 22 libraries, on average, only 14% of the total expression level was assigned to the overlapping TSS. In other cases, such as *ANAPC16* and *ASCC1,* the expression from overlapping TSSs is dominant. In many instances, however, overlapping TSSs are minor in some samples and major in others. A good example is the pair of genes *PNKD* and *AAMP.* In this case, overlapping and nonoverlapping TSSs were used together in 55 libraries. In 16 samples, the expression from nonoverlapping TSS was higher, and in 39 libraries, expression from overlapping TSS dominated. Of these, in 14 cases, more than 80% of the total expression was initiated in overlapping regions (source: OverGeneDB database^50^).

While Karlsson et al.^32^ demonstrated, based on single-cell studies, that TSSs are generally regulated by common factors, Batut et al.^47^ determined that alternative promoters generally implement distinct regulatory programs. Our studies and analysis of transfected and nontransfected cell lines appear to support the second scenario, since the switch to upstream promoters could result from changes in transcription factor expression levels in response to transfection. This finding suggests that alternative promoters may not be regulated by the same factors. In addition, analysis of transcription factor binding sites demonstrated that in many cases, binding sites for specific factors were detected only in the proximity of one alternative TSS. Nevertheless, the degree of changes in TSS usage between cell lines may reflect cellular robustness against transfection or a generally changing environment, but further research is warranted to explain the mechanisms responsible for the regulation of alternative TSS usage.

Previous studies have suggested that the sense-antisense overlap between genes may have a regulatory role, including downregulation of gene expression^28^. One of the proposed mechanisms of expression regulation is transcription interference via polymerase collision or by blocking polymerase binding sites^14^. It was suggested that overlapping genes have negatively correlated expression^28^. However, we found only one pair of genes with a negative correlation expression in libraries where these genes overlap. Out of 73 gene pairs for which the expression correlation was studied, 21 pairs had a positive expression correlation while they overlapped, and four pairs had a positive expression correlation while the genes were nonoverlapping. In addition, the expression levels of eight pairs were positively correlated, regardless of whether the genes overlapped. This result suggests that the head-to-head oriented overlap may be activating, rather than repressing, and supports results from other studies^10^. A positive correlation could be explained by the presence of bidirectional promoters that regulate many gene pairs in a head-to-head orientation^51^, which results in frequently observed concordant expression. Moreover, the activity of bidirectional promoters was observed to be regulated at different points during transcription, which gives rise to diverse types of transcripts^52^.

It is feasible that transcriptional interference may depress the expression level of both involved genes. Our studies, however, show the opposite effect of gene overlap. Genes from the abovementioned 73 pairs had significantly higher expression when utilizing overlapping TSSs. In many instances, an overlapping TSS was employed in addition to the nonoverlapping TSS. Utilizing multiple TSSs could create a reservoir of RNA polymerase, which would facilitate rapid activation of one or both genes and increase the transcription level. Nevertheless, the difference in the expression level was significant, regardless of the number of active TSSs. Moreover, when genes utilized nonoverlapping TSSs, their average expression was significantly lower than when only one gene from a pair was expressed. This observation is intriguing and, at present, difficult to explain. It is possible that bidirectional promoters, utilized when genes are not overlapping, are less robust than more distal and gene-specific promoters, since they have to serve two genes at the same time.

The fact that overlapping genes do not exhibit a decrease in the expression level due to possible transcriptional interference suggests that there are mechanisms preventing such consequences of this gene arrangement. One feasible scenario could be independent expression of overlapping genes from homologous chromosomes. The results of our analysis indicated that monoallelic expression does not occur more frequently in the case of overlapping genes. However, for the majority of genes, we could not establish the expression pattern due to lack of polymorphic sites. Moreover, monoallelic expression can be random, and genes can be expressed either from the paternal or maternal allele in different cells^53, 54^. Therefore, the signal from multiple cells may indicate biallelic expression, while at the cell level, the expression is actually monoallelic. For the abovementioned reasons, we cannot exclude this mechanism as one of factors that helps to impede transcriptional interference.

## Conclusions

Our knowledge of TSS usage has increased dramatically in recent years. Transcriptome-wide studies have shown that TSS use is highly tissue-specific and that alternative TSS usage is more common among genes involved in development. Alternative TSS usage can affect protein diversity by altering N-terminal polypeptides and can cause differences in translation productivity. Therefore, it appears likely that alternative promoters evolved to produce the required mRNA isoforms in the correct tissue and at the correct time. Studies performed by our group demonstrate for the first time that alternative TSSs may have an additional function, i.e., switching gene organization from nonoverlapping to overlapping and vice versa. These functions could play a regulatory role at the transcriptional level via transcriptional interference and at the posttranscriptional level by forming double-stranded RNA. Our studies did not confirm that overlap between genes caused downregulation of their expression. Analyses also showed that the overlap between genes is not a stable feature and depends on which TSSs are utilized in a given cell type or at the particular state of a cell. Permanent utilization of overlapping promoters can be attributed only to a small number of head-to-head oriented gene pairs. This phenomenon needs to be further investigated to determine whether such genomic organization of protein-coding genes indeed has functional meaning and how these genes ‘escape’ from transcriptional interference. Mechanisms other than monoallelic expression, such as mutually exclusive expression at a given time point, are currently being investigated in our laboratory.

## Materials and methods

### Transcription start site and gene location data

The positions of the 47,912 annotated RefSeq transcripts were obtained from the UCSC Genome Browser^55^ assembly hg38. The collection of alternative transcription start sites (TSS) corresponding to the RefSeq transcripts in several dozen human organs and cell lines was received from the Database of Transcriptional Start Sites (DBTSS) release 8 and 9^56, 57^. A total of 73 human libraries from the DBTSS were used. All libraries are listed in Supplementary Table S1.

TSS data were further filtered according to the following criteria: (1) only TSSs described as confident in DBTSS database were taken under consideration; (2) TSSs with expression levels lower than 5 ppm (parts per million) were rejected, as suggested by Yamashita^35^; and (3) the maximum distance between the TSS position and the associated gene start was limited to 5 kb from the annotated 5’ end of the given gene to limit false positive TSS assignments.

### Overlapping gene identification

Overlapping genes were identified separately in each library based on the coordinates of the most upstream TSS assigned to each gene. Genes were considered “overlapping” in a particular library if they shared at least 1 bp at their 5’ ends.

### Differential gene expression analysis

Differential gene expression analysis was conducted for four transfections with STAT6 silencing siRNA and nontargeting siRNA libraries (*BEAS-2B IL4- STAT6 siRNA, BEAS-2B IL4* + *STAT6 siRNA, BEAS-2B IL4- control siRNA* and *BEAS-2B IL4* + *control siRNA*) and two untransfected libraries (*BEAS-2B IL4- parent, BEAS-2B IL4* + *parent*), with the latter considered in this study as a control for transfection. The analysis was conducted using the edgeR program^58^ based on raw TSS-Seq read counts summed at the gene level. As differentially expressed, we considered all genes whose log_2_(fold-change) value was higher than 1.5 or smaller than − 1.5 and whose FDR score was smaller than 0.05.

### Analysis of transcription factors

A set of differentially expressed transcription factors was acquired by intersection of all differentially expressed genes with a list of 2454 human transcription factors from the UniProtKB database^59^, accessed at January 20th 2019, with the following query: *"transcription factor" AND reviewed:yes AND organism:"Homo sapiens (Human) [9606] "*. Identification of the binding sites was conducted with TFBSTools (version 1.14.0)^60^. The required was a significance score of at least 95% using 367 human TFs as a reference, which were downloaded in PFM format from the JASPAR database^36^. For the TSS to become associated with a TF, its binding motif needed to be located up to 500 bp from the transcription start site.

### Statistical analysis

Statistical analysis was performed using R and Python programming languages. Hierarchical clustering of genes and libraries was performed using the “ward” method from the SciPy package. It was performed on a two-dimensional matrix representing all genes (x axis) overlapping in at least one library (y axis), with 0 standing for no expression, 0.5 in case of expression without overlap, and 1 in case a particular gene utilized at least one overlapping promoter to initiate transcription in a particular library.

### Allele-specific expression

Whole genome sequencing (WGS) and RNA-seq data sets from 22 adenocarcinoma samples^57^ were used for analysis of allele-specific expression. Primers, adapters, and low-quality reads from WGS and RNA-seq data were removed with Trimmomatic-0.36^61^, followed by FastQC^62^ for quality control. In the case of RNA-seq bowtie2 (version 2.2.3)^63^, was additionally used to remove reads representing rRNA. Reads were mapped to the reference genome hg38. To this end, BWA MEM (version 0.7.10)^64^ and STAR2 (v 2.5)^65^ were used to map WGS and RNA-seq reads, respectively. PCR duplicates were marked with Picard (version 2.9.2)^66^. Gene expression was estimated using StringTie (version 2.0)^67^ and a minimum of 5 FPKM was required to classify a gene as expressed.

RealignerTargetCreator tool was used to realign WGS data to genetic variants from dbSNP database^68^, containing a collection of single nucleotide polymorphisms (SNPs), followed by base quality score recalibration performed with BQSR; both tools from GATK (version 3.7)^69^. SNPs in the DNA sequence were identified with HaplotypeCaller (GATK), and the results were filtered by VariantFiltration (GATK) with standard parameters. VCF files containing high-quality variant calls were used to realign RNA-seq reads utilizing RealignerTargetCreator and BQSR. Next, RNA-seq read counts per allele were computed with ASEReadCounter (GATK) with following parameters: -U ALLOW_N_CIGAR_READS -minDepth 10—minMappingQuality 20—minBaseQuality 2.

Expressed genes with heterozygous SNPs were classified as (1) monoallelic, when at least 98% of the reads were mapped to one allele and less than 2% of the reads were mapped to the second allele; (2) with skewed expression, when less than 20% of the reads were mapped to one allele and at least 2% of the reads were mapped to second allele; (3) biallelic expression, when at least 20% of the reads were mapped to both alleles. To classify genes as monoallelic, skewed or biallelic, only SNPs located outside the overlapping region were considered. Genes with conflicting signals from different SNPs were marked as inconclusive and excluded from analysis.

Copy number variation was called using CNVnator version 0.4^70^. We adjusted the value of the parameters hist, stat, and call for read depth (RD) to 4 and filtered VCF records for deletion and duplication hits. Genes encoded in nondiploid genome regions were excluded from analysis.

### Functional enrichment analysis

Pathway enrichment analysis was conducted on grouped lists of overlapping genes (e.g. adult tissues, fetal tissues etc.) using Enrichr^71^. The following reference libraries were utilized: KEGG_2016, BioCarta_2016, WikiPathways_2016, Reactome_2016, GO_Biological_Process_2018, GO_Cellular_Component_2018, GO_Molecular_Function_2018, KEA_2015. The overlap of at least 4 genes between query list of genes and pathway from reference library was required to include the gene signature into the analysis. Lists of analyzed pathways and their corresponding *p*-values and corrected *p*-values (FDR score) were then extracted, converted to − log_10_(*p*-value) and − log_10_(FDR) value, merged into one table (Supplementary Table S5), and sorted in descending order by the sum of − log_10_ converted values in all groups of overlapping genes associated with particular gene signature.

## Supplementary Information

Supplementary Information

## Data Availability

The datasets generated and/or analyzed during the current study are available in the OverGeneDB database (http://overgenedb.amu.edu.pl)^50^ and supplementary information file.
